# Interaction of surface pattern and contour shape in the tilt after effects evoked by symmetry

**DOI:** 10.1038/s41598-021-87429-y

**Published:** 2021-04-13

**Authors:** Ko Sakai, Yui Sakata, Ken Kurematsu

**Affiliations:** Computational Vision Science Laboratory, Department of Computer Science, University of Tsukuba, 1-1-1 Tennodai, Tsukuba, Ibaraki 305-8573 Japan

**Keywords:** Visual system, Psychology

## Abstract

Integration of multiple properties of an object is a fundamental function of the visual cortex in object recognition. For instance, surface patterns and contour shapes are thought to be crucial characteristics that jointly contribute to recognition. However, the mechanisms of integration and corresponding cortical representations have not been fully clarified. We investigated the integration of surfaces and shapes by examining the tilt after effects (TAEs) evoked by the symmetry of patterns and contours. As symmetry in both pattern and contour evokes TAEs, we can directly measure the interaction between the two. The measured TAEs exhibited mutual transfer between the symmetry of the pattern (SP) and that of the contour shape (SS), i.e., adaptation by SP (SS) evoked TAEs when tested by SS (SP), suggesting the existence of an integrated representation. Next, we examined the interaction between SP and SS when both were simultaneously presented in adaptation. Congruent adaptors wherein their symmetry axes aligned evoked compressive interaction, whereas incongruent adaptors wherein the axes of SP and SS tilted to the opposite directions evoked subtractive interaction. These results suggest the existence of a cortical representation that integrates the properties of the surface and shape with suppressive interactions, which can provide crucial insights into the formation of object representation as well as the integration of visual information in the cortex.

## Introduction

Robust and reliable object recognition is a remarkable feature of the visual system. Objects are usually characterized by multiple attributes, including shape, color, texture, motion, and dimension. The visual system takes advantage of integrating multiple attributes to establish robustness and reliability. Surface properties such as color, texture, and glossiness and object shapes such as contours of the whole and parts are key features for object recognition. Recent psychophysical studies have addressed the joint contribution of surfaces and shapes, such as a surface-based representation of a shape^1^ and a crucial role of surface saliency in object recognition based on contour shapes^2^. The cortical representation of surface properties^3–5^ and object shape^6,7^ has been reported in intermediate- to high-level ventral visual cortices. Conversely, recent electrophysiological studies have reported the dissociation of neural coding for surface properties and contour shape in the intermediate-level visual cortex of monkeys^8–10^; specifically, many neurons in visual area V4 were found to be selective for textures and shapes, but their encoding was largely independent across neurons. The dissociation of texture and shape has also been reported using event-related potentials (ERPs)^11^. It is crucial to clarify how surfaces and shapes interact and are integrated and what cortical representations take place through the construction of object recognition.

A variety of symmetries, such as symmetry in pattern, texture, and shape, are ubiquitous in both natural and artificial objects and contribute to their recognition. Symmetry in pattern and texture contributes to perceptual organization, figure-ground segmentation, and shape detection, which leads to the detection of single objects e.g.,^12^; for review^13,14^. The perceptual properties that are consistent with the detection of single objects have been reported in the symmetry of dot patterns^15–19^, regular patterns^20^, and natural textures^21^. Imaging studies have indicated that early- to intermediate-level ventral visual cortices, such as V3, V4, VO1 and LOC, respond to various types of symmetry in pattern and texture^22,23^, with quantitatively different activities for different types of symmetry^14^. Moreover, symmetry in contour shape directly contributes to shape perception. The medial axis, a group of local symmetry axes, has been a prominent candidate for shape coding e.g.,^24–28^. Intermediate-level visual cortices such as V4 are considered crucial for the detection of shape symmetry^29^. Psychophysical studies have also reported that contour symmetry enables rapid shape detection^30^. However, the relationship between symmetry in pattern/texture and shape as well as their representation in the visual cortices has not been fully clarified. Investigations on the symmetry in pattern and shape are expected to provide crucial evidence for the integration and representation of surfaces and shapes in intermediate-level visual processing. Psychophysical studies have reported adaptation to a symmetry axis e.g.,^31–34^. Tilt after effects (TAEs) with respect to a symmetry axis were observed with adaptation to a variety of symmetry types, such as dot patterns^35^, natural stimuli^36^, and contour shapes^37^. Recent studies have investigated various properties of symmetry based on the detection of symmetry axes and TAEs^35,36,38–40^. As symmetry in both pattern and contour shape evokes adaptation to symmetry axes, TAEs are expected to be a good measure to examine the interaction and integration of surface patterns and contour shapes.

**Figure 1:**
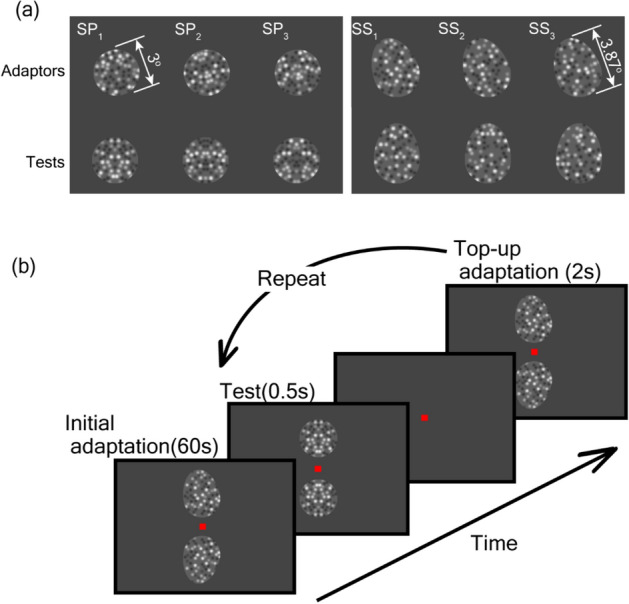
(**a**) Examples of stimuli with symmetry of pattern (SP; left) and shape (SS; right). In these examples, the symmetry axes of the adaptors (the top row) were tilted − 10° (anticlockwise) from the vertical direction, while those of the tests (the bottom row) were vertical. The numbers in white on the top left of each stimulus identify distinct dot patterns. In the stimuli with SP, the corresponding adaptor and test (sharing the same column) had the same dot pattern, but their luminance contrast was inverted to avoid influence from the contrast. In the stimuli with SS, dot patterns were random and different between the adaptor and test. (**b**) An illustration of the experimental procedure. A pair of adaptation stimuli were shown for 60 s, and a pair of test stimuli were then presented for 0.5 s. The stimulus pairs consisted of the same stimulus with their symmetry axes tilted in the opposite directions. In this example, the adaptors on the top and bottom were tilted anticlockwise (–θ) and clockwise (+ θ) from the vertical direction, respectively. The participants were asked to indicate which stimulus was tilted clockwise from the vertical during the presentation of a blank screen. Immediately after the answer, a pair of top-up adaptors were presented for 2 s. The trial was repeated 25 times using the staircase method.

We psychophysically investigated the cortical representation that integrates the properties of the surface and shape of an object and the interaction between the two in the formation of the representation. Specifically, we focused on TAEs of symmetry axes evoked by symmetry of pattern (SP) and contour shape (SS). We hypothesized that two neural circuitries contributing to the perception of SP and SS operate independently and that they contribute to the formation of the integrated representation. First, we examined whether the TAEs evoked by SP and SS transfer between them, which would provide crucial evidence for the integrated representation. In the psychophysical experiment, participants were adapted to SP and SS and tested with stimuli that consisted of SS and SP, respectively (Fig. 1). The results revealed the partial mutual transfer of TAEs between SP and SS; that is, TAEs were observed when participants were adapted to SS and tested by SP, and vice versa, suggesting the existence of an integrated representation that combines the properties of surface and shape.

We further investigated the interactions between SP and SS in the adaptation. First, we examined the interaction evoked by antagonistic, *incongruent adaptors* wherein SP and SS were presented simultaneously with their symmetry axes tilted in opposite directions with respect to the reference (P–S adaptor). After adaptation to the incongruent stimuli, we measured TAEs by the test stimuli with SP (P test stimuli) and that with SS (S test stimuli) along with the congruent test stimuli wherein SP and SS were presented simultaneously with their symmetry axes aligned (P + S test stimuli). The measured TAEs exhibited a subtractive interaction between SP and SS. Specifically, the magnitudes of TAEs with the P + S test stimuli were between those with the S and P test stimuli, suggesting a subtractive or suppressive interaction between SP and SS. Next, we examined the interaction evoked by synergic, *congruent adaptors* that comprised SP and SS with their symmetry axes aligned (P + S adaptor). TAEs were measured with the P, S, and P + S test stimuli to reveal how SP and SS interact with each other when they are congruent. With the P and S test stimuli, TAEs evoked by P + S adaptors exhibited similar magnitudes to those evoked by P and S adaptors, respectively. Since the P + S test stimulus included both SP and SS, it was expected to exhibit the greatest magnitude of TAEs with the P + S adaptors. However, the magnitudes of TAEs with the P + S test stimuli were not the greatest among those with the three types of test stimuli. These results indicate compressive interaction between SP and SS; even when both SP and SS exist simultaneously in congruence, they are not processed additively to avoid overestimation. The subtractive and compressive interactions observed with the incongruent and congruent adopters, respectively, suggest suppressive mutual interactions through the formation of an integrated representation. These results support the hypothesis that SP and SS are processed independently and that an integrated representation follows through interactions. The results also suggest a cortical representation that integrates the properties of surface and shape, providing crucial insights into the formation of the representation of objects and the integration of information in the cortex.

## Results

### Transfer of adaptation between pattern and shape symmetry (Exp. 1)

**Figure 2:**
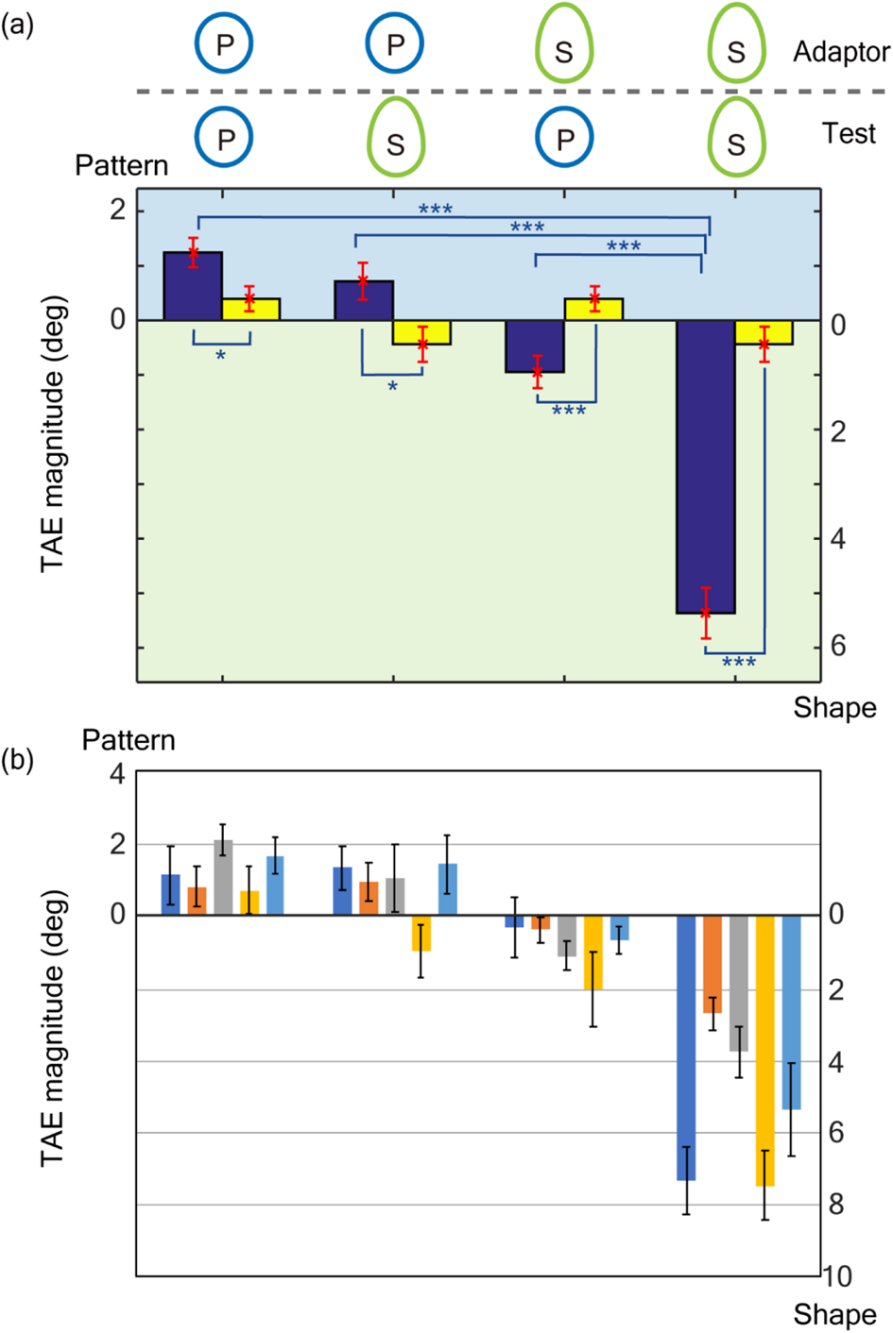
Measured TAE magnitudes, including transfer conditions (Exp. 1). (**a**) Mean magnitudes across the participants. Icons on the top indicate the combination of adaptor and test types (P and S denote symmetry in pattern and shape, respectively). Blue and yellow bars show the measured TAEs for the four conditions (from the left, TAE_P,P_, TAE_P,S_, TAE_S,P_, and TAE_S,S_) and those for the corresponding blank conditions (TAE_B_), respectively. The TAE with the adaptation to SP is plotted toward the positive direction in the ordinate (the scale on the left), whereas the TAE to SS is plotted toward the negative direction (the scale on the right) to clarify the transfer between SP and SS. (**b**) The measured magnitude of each participant. Colors indicate individuals. Error bars show the standard error (SEM), and asterisks denote statistical significance (*p < 0.05; **p < 0.01; ***p < 0.001).

We investigated the cortical representation that integrates the properties of the surface and shape of an object with psychophysical experiments that examined the transfer of adaptation between SP and SS. Specifically, in the experiment, participants were adapted to SP and SS and tested with stimuli that consisted of the other type of symmetry, SS and SP, respectively. Adaptation transfer would suggest the existence of the integrated representation that consisted of a group of neurons. We measured TAEs in the transfer conditions where the adaptor and test assumed different types of symmetry (TAE_P,S_: TAE observed with P adaptor and S test; TAE_S,P_: S adaptor and P test), and in the control conditions where both adaptor and test were SP (TAE_P,P_) or SS (TAE_S,S_) (refer to the top of Fig. 2 for the combinations of the adaptors and tests). The adaptors were rotated along imaginary circles of radius 1 deg. (degree in visual angle) with the tilt of their symmetry axes remaining constant to prevent adaptation to low-level image properties, such as luminance contrast and local edges (refer to Procedure and Apparatus for details). The mean measured TAE magnitudes across participants in the control conditions, TAE_P,P_ and TAE_S,S_, were 1.25° and 5.36°, respectively, and those in the transfer conditions, TAE_P,S_ and TAE_S,P_, were 0.72° and 0.95°, respectively (Fig. 2). As the participants could perceive a slight tilt in the test stimuli without adaptation, we also measured the perceived orientation of the test stimuli (blank condition, TAE_B_; refer to Supplementary note S1 for details) and tested whether the measured TAEs were significantly different from those in the blank condition. Pairwise *t*-tests showed significant effects in all four conditions (TAE_P,P_: t(59) = 2.19, p = 0.032; TAE_P,S_: t(59) = 2.39, p = 0.020; TAE_S,P_: t(59) = 3.57, p < 0.001; TAE_S,S_: t(59) = 9.10, p < 0.001 (p < 0.001 with randomization test)). The significant magnitudes in the control conditions (TAE_P,P_ and TAE_S,S_) indicate that our stimuli indeed evoked TAEs by adapting to symmetry to a similar degree as that reported in previous reports^32,35^. The significant magnitudes in the transfer conditions (TAE_P,S_ and TAE_P,S_) indicate that the adaptation to the symmetry axis of pattern and contour shape altered the perception of the symmetry axis of contour shape and patterns, respectively, indicating the mutual transfer between SP and SS. This mutual transfer suggests a cortical representation that integrates SP and SS.

The mean magnitude of TAE_S,S_ was greater than that of the others. A repeated-measure ANOVA with Greenhouse–Geisser (GG) correction for violation of sphericity showed significance of condition (F(3,177) = 36.8, η^2^ = 0.336, p < 0.001), and post hoc tests with Bonferroni correction showed that the magnitude of TAE_S,S_ was significantly greater than those of the other conditions (TAE_S,S_ > TAE_P,P_ = TAE_P,S_ = TAE_S,P_; refer to Supplementary Table S2-1 for details), indicating the greater effects of SS than SP. The magnitude of TAE_S,S_ was consistent with that reported previously, which were measured with symmetric dot patterns surrounded by symmetric contours^35^. SS tended to evoke a greater TAE than SP, at least with our stimulus configurations.

The S stimuli were asymmetric dot patterns within an egg-shaped contour. It could be argued that the particular dot patterns and contour shapes evoked the adaptation. To rule out such possibilities, we performed experiments that controlled the inner dots and contour shapes. Specifically, we tested stimuli without inner dots and the alternative presentation of upright and upside-down egg-shaped stimuli for adaptation. These stimuli did not evoke significantly different TAEs from the original stimuli (TAE_S,S_), indicating that our experiment indeed measured the TAEs evoked by SS (refer to Supplementary note S3).

The mutual transfer between SP and SS suggests an integrated representation of the two. Detailed discussions on the neural mechanisms are provided in the Discussion section and Supplement S5a. To further investigate the interaction between SP and SS, we measured TAEs with novel adaptors wherein SP and SS were given independently in opposite directions, as described in the following section.

### Antagonistic interaction between pattern and shape symmetry (Exp. 2)

**Figure 3:**
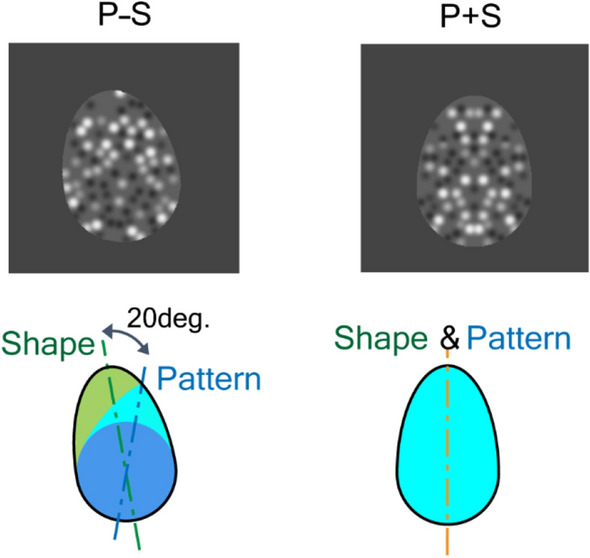
Left: An example of incongruent, antagonistic stimuli (P–S stimulus) in which the axes for SP and SS are tilted in opposite directions. The dot pattern is mirror symmetric with respect to the axis of SP, which is 10° (clockwise from the vertical) in this example. The egg-shaped contour of the P–S stimulus is also mirror symmetric with respect to the axis of SS, which is − 10° in this example. Right: An example of congruent, synergic stimuli (P + S stimulus) in which the axes for SP and SS are tilted in the same direction (the vertical in this example). The stimulus was generated in the same way as P–S stimuli except for the tilt of the symmetry axes.

**Figure 4:**
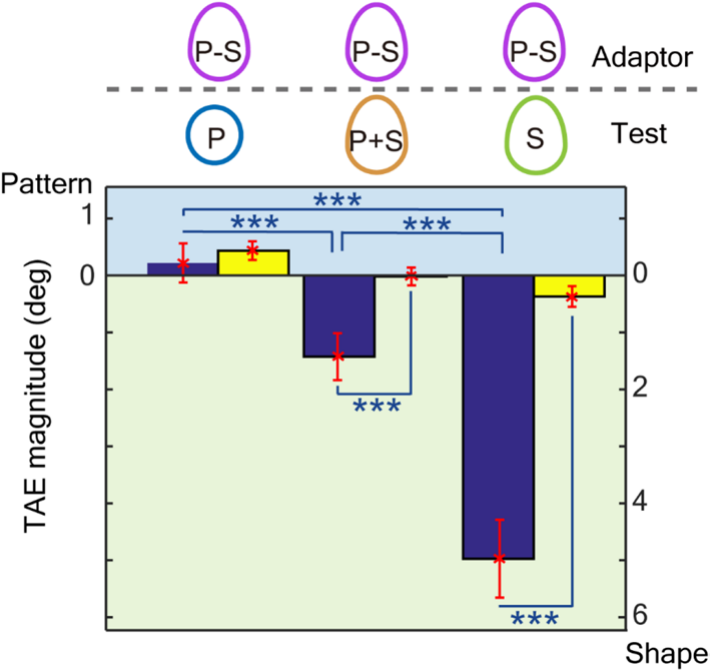
The mean TAE magnitudes across the participants with incongruent, antagonistic adaptors (Exp. 2). The same conventions are used as in Fig. 2. Blue and yellow bars show the measured TAEs for the three conditions (from the left, TAE_P–S,P_, TAE_P–S,P+S_, and TAE_P–S,S_) and those for the corresponding blank conditions, respectively. The TAE for SP is plotted toward the positive direction in the ordinate (the scale on the left), whereas the TAE for SS is plotted toward the negative direction (the scale on the right). Error bars show the standard error (SEM), and asterisks denote statistical significance (*p < 0.05; **p < 0.01; ***p < 0.001).

We further examined the interactions between SP and SS. Specifically, we focused on antagonistic interactions evoked by incongruent adaptors wherein SP and SS were presented simultaneously with their symmetry axes tilted in opposite directions (–θ and + θ, respectively; refer to P–S in Fig. 3). With this configuration of stimulus, the participants were adapted to SP and SS simultaneously in opposite directions. We measured TAEs with congruent test stimuli wherein SP and SS were presented simultaneously with their symmetry axes aligned (P + S test stimuli; refer to P + S in Fig. 3), as well as the P and S test stimuli. Since the directions of adaptation for SP and SS were opposite, the TAE direction and magnitude provide a clue on the effects of antagonistic adaptation by SP and SS. First, we consider the cases with the P and S test stimuli. With the P test stimulus, we expect a TAE corresponding to SP, which is in the direction of + θ. Alternatively, with the S test stimulus, we expect a TAE in the opposite direction (–θ). Now, we consider the case with the P + S test stimulus where SP and SS interact with each other. If SP were dominant over SS and exclusive, we would expect a TAE in the direction of + θ. Alternatively, if SS were dominant and exclusive, we would expect a TAE in the opposite direction (–θ). Finally, if SP and SS interacted subtractively or antagonistically, we would expect a TAE between –θ and + θ depending on the strength of SP and SS. With the incongruent adaptors, we measured TAEs by the P, S, and P + S test stimuli to examine the interaction between SP and SS (refer to the top of Fig. 4).

The adaptors had the axes of SP and SS tilted 10° clockwise and anticlockwise from the vertical, respectively, or vice versa (anticlockwise and clockwise, respectively; P–S in Fig. 3). With the adaptors always P–S, we measured TAEs using the P, S and P + S test stimuli. The mean measured TAE magnitudes across participants were 0.22° with the P test stimuli (TEA_P–S,P_), 1.43° with the P + S test (TEA_P–S,P+S_), and 4.97° with the S test (TEA_P–S,S_) (Fig. 4; refer to Supplementary note S4 for individual data). The TAEs with the P + S and S test stimuli were significantly greater than TAE_B_ (trials without adaptation), while the TAE with the P test stimuli was not (pairwise *t*-test; refer to Supplementary Table S2-2). The TAE with the P + S test stimuli lay between the TAEs from the P and S tests. A repeated-measure ANOVA with GG correction for violation of sphericity showed significance of the condition (F(2,118) = 64.4, η^2^ = 0.345, p < 0.001), and post hoc tests with Bonferroni correction showed significant differences among conditions (TAE_P–S,P_ < TAE_P–S,P+S_ < TAE_P–S,S_; refer to Table S2–3). The magnitude of the TAE with the P + S test stimuli lay between the magnitudes of the TAEs with the P and S tests, suggesting a subtractive interaction between SP and SS. The subtraction can be attributed to the cancelation between TAEs evoked by SP and SS. The greater magnitude for the TAE with the S test compared to that with the P test indicates that the adaptation to SS is dominant over that to SP, which is consistent with the result in the previous section. The bias of the TAE with the P + S test toward SS also indicates the dominance of SS over SP.

Another prominent characteristic of the result for incongruent adaptation is that the magnitude of TAE with the S test stimuli was similar to that observed after adaptation to SS in Exp. 1 (TAE_P–S,S_ = TAE_S,S_); pairwise t-test, t(59) = 0.613, p = 0.542). Since the adaptation was incongruent where the symmetry axes were tilted in the opposite directions for SP and SS, SP and SS could cancel each other; therefore, a small degree of TAE can be expected. This characteristic provides crucial evidence for understanding the underlying neural mechanisms. Specifically, the result suggests an independent representation of pattern and shape so that adaptation in the shape representation is not affected by the adaptation to SP. Detailed discussions on the neural mechanisms are provided in the Discussion section and Supplement S5b.

Alternatively, with the P test, the TAE magnitude was smaller than that observed after adaptation to SP (TAE_P–S,P_ < TAE_P,P_; pairwise t-test, t(59) = 2.33, p = 0.023), which is inconsistent with the cases using the S test (TAE_P–S,S_ = TAE_S,S_). The smaller magnitude of TAE_P–S,P_ might have originated from the nature of the incongruent adaptor in which approximately 25% of the dots did not have matched pairs, as illustrated in Fig. 3 (the green region in the figure). Since the extent to which the symmetric pattern that was included in the P–S adaptor (light-blue plus blue regions) was approximately 20% larger than that in the P adaptor (a circular region with blue), the effect of SP in the P–S adaptor was greater than in the P adaptor. However, it is possible that the asymmetric region could disrupt the effect, and thus, this nature of the P–S adaptor might account for the small magnitude observed with the P test. Further discussion on the differences in the SP and SS effects is provided in the Discussion section. In the present section, we examine the antagonistic interaction between SP and SS with incongruent adaptation, in which the symmetry axes of patterns and contour shapes are tilted in opposite directions. In the next section, we examine the synergic interaction with the congruent adaptation in which the symmetry axes are aligned and tilted in the same direction.

### Synergic interaction between pattern and shape symmetry (Exp. 3)

We investigated synergic interactions evoked by congruent adaptors wherein SP and SS were presented simultaneously with their symmetry axes aligned and tilted in the same direction. Since the previous section suggested a linear interaction between SP and SS, the TAE was expected to double with the congruent adaptor for any of the test stimuli. However, this prediction appears to be doubtful, as we do not observe extra tilt with the congruent condition that is common across many natural and artificial objects. Rather, compressive interaction between SP and SS is expected; the magnitude of the TAE with the congruent adaptors is expected to be no greater than those with SP and SS adaptors. We performed the experiments with the procedure and test stimuli identical to the experiment described in the previous section. The congruent adaptor had the same pattern and shape as the P + S test stimuli, but the contrast of dots was inverted to cancel out the influence of luminance. The test stimuli were the P, S, and P + S test stimuli.

**Figure 5:**
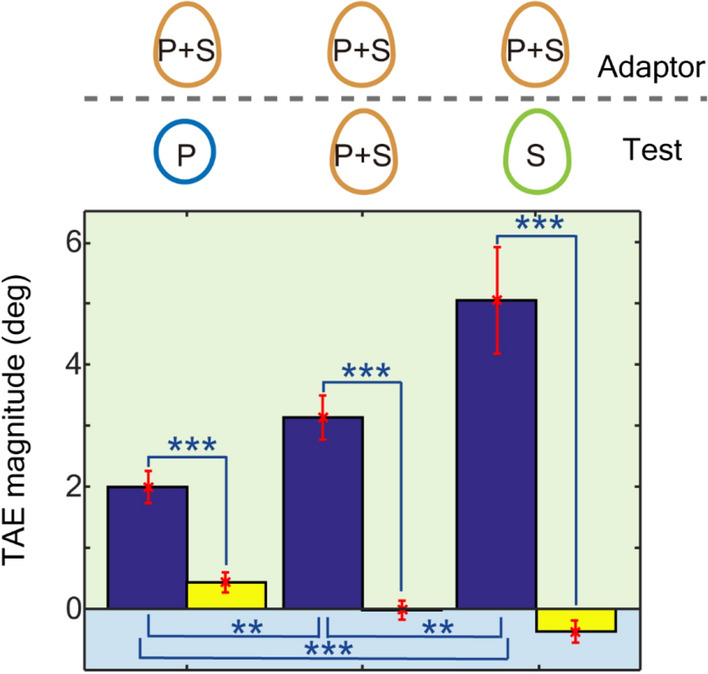
The mean TAE magnitudes across the participants with congruent, synergic adaptors (Exp. 3). The same conventions are used as in Figs. 2 and 4. Blue and yellow bars show the measured TAEs for the three conditions (from the left, TAE_P+S,P_, TAE_P+S,P+S_, and TAE_P+S,S_) and those for the corresponding blank conditions, respectively. Error bars show the standard error (SEM), and asterisks denote statistical significance (*p < 0.05; **p < 0.01; ***p < 0.001).

The mean measured TAE magnitudes across participants were 2.00° with the P test stimuli (TAE_P+S,P_), 3.13° with the P + S test stimuli (TAE_P+S,P+S_), and 5.05° with the S test stimuli (TAE_P+S,S_) (Fig. 5; refer to Supplementary note S4 for individual data), all of which were significantly greater than TAE_B_ (the blank condition; pairwise *t*-test; refer to Supplementary Table S2-4). The TAE with the P + S test stimuli lay between the TAEs from the P and S tests. A repeated-measure ANOVA with GG correction for violation of sphericity showed significance of condition (F(2,118) = 17.8, η^2^ = 0.155, p < 0.001), and post hoc tests with Bonferroni correction showed significant differences among conditions (TAE_P+S,P_ < TAE_P+S,P+S_ < TAE_P+S,S_; refer to Table S2–5). The smaller magnitude of the TAE with the P + S test stimuli compared to that with the S test (TAE_P+S,P+S_ < TAE_P+S,S_) indicates the compressive interaction between SP and SS. The greater magnitude of the TAE with the P + S test stimuli compared to that with the P test (TAE_P+S,P+S_ > TAE_P+S,P_) might be due to the dominance of SS over SP that was observed in the previous sections. If the strengths of SS and SP were similar, we would observe a greater magnitude in TAE_P+S,P_ than in TAE_P+S,P+S_.

Another prominent characteristic of the result for congruent adaptation is that the magnitudes of TAEs were not greater than those observed after adaptation to SS or SP in Exp. 1 (TAE_P+S,S_ = TAE_S,S_; TAE_P+S,P_ = TAE_P,P_; pairwise *t*-test, TEA_P+S,S_: t(59) = 0.462, p = 0.646; TEA_P+S,P_: t(59) = 1.81, p = 0.075). Since the adaptation was congruent where the symmetry axes were tilted in the same direction for SP and SS, SP and SS could enhance each other; therefore, a greater degree of TAE could be expected. This prominent characteristic suggests mutual suppression between SP and SS. Without suppression, the degree of adaptation would be doubled, or at least increased, compared to the cases with adaptation to SP or SS. Mutual suppression reduces adaptation only when the adaptor includes SP and SS with the same tilt, whereas suppression does not work when the adaptor is comprised of either SP or SS (or their tilts are different), leading to the simultaneous reproduction of the linear and compressive operations in incongruent and congruent conditions, respectively. Further discussions on the neural mechanisms are provided in the Discussion section and Supplement S5c.

The results of these three experiments suggested an integrated representation of symmetry and mutual suppression between the pattern and shape symmetry. Mutual suppression works similarly to the logical disjunction (OR) operation when SP and SS exist simultaneously such that overrepresentation is avoided. This compressive nonlinearity appears to be ecologically plausible because it correctly determines the orientation of an object in which SP and SS provide the same axis of symmetry, as is often observed in natural and artificial objects.

## Discussion

To understand how the shape and surface properties of an object are integrated to form the representation of the object, we investigated the interaction between symmetry of shape (SS) and symmetry of pattern (SP). We set up psychophysical experiments in which the TAEs evoked by SP and SS were measured simultaneously. The results showed partial mutual transfer of the adaptation between SP and SS, suggesting the presence of a cortical representation that integrates SP and SS. We further investigated the interactions between SP and SS in the adaptation. Antagonistic adaptation, wherein the symmetry axes of SP and SS were tilted in opposite directions, showed subtractive interaction between the two. In contrast, synergic adaptation, wherein the symmetry axes were tilted in the same direction, showed compressive interaction. These results suggest compressive nonlinearity in the integration; TAEs are processed linearly when single or incongruent adaptors are presented but compressively when congruent adaptors are presented. This nonlinearity is ecologically advantageous, as the overestimation of tilt is avoided; we do not see that the tilt doubles even when SP and SS are congruent. These results support the notion that the neural mechanisms underlying the SS and SP operate independently, followed by the cortical representation that integrates the SP and SS with mutual suppression.

**Figure 6:**
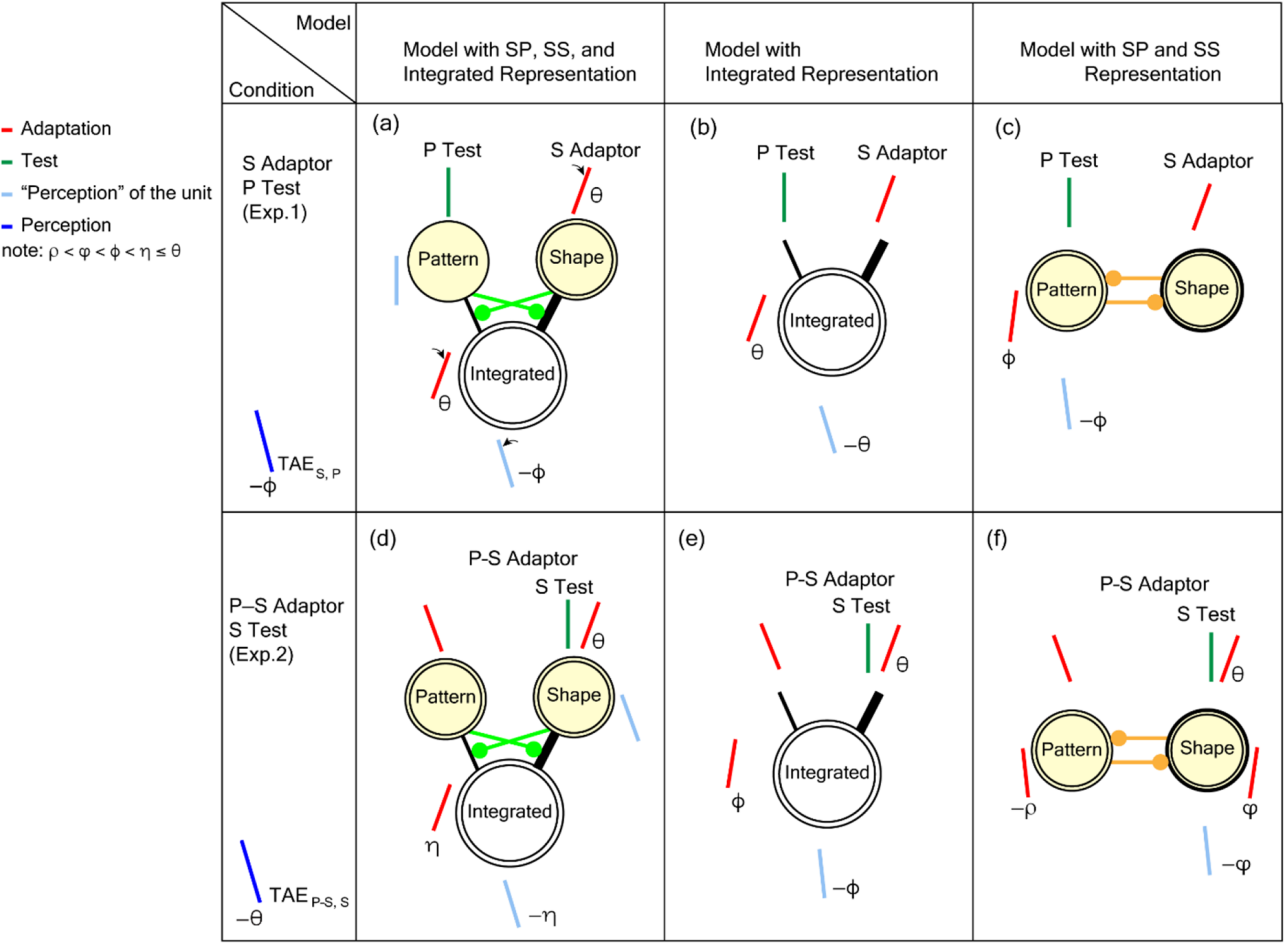
A comparison of the conceptual model. The left column (panels **a**,**d**) indicates the behavior of the proposed model with the independent representations of SP and SS, followed by the integrated representation, as indicated by "Pattern", "Shape", and "Integrated", respectively. The double circles indicate that these circuitries were adapted. The center column (panels **b**,**e**) indicates an alternative model with the integrated representation but without independent pattern and shape representations. The right column (**c**,**f**) indicates another model comprised of the pattern and shape representations with mutual facilitatory connections. The top row indicates the model responses for the transfer condition with the S adaptor and P test stimulus (TAE_S,P_). The bottom row indicates the responses for the incongruent condition with the S test (TAE_P–S,S_). Throughout the panels, red and green bars on the top illustrate the tilt of the adaptor and test stimulus, respectively. Red and light-blue bars around the representations illustrate the hypothetical adaptation (i.e., neurons responsive to this tilt are activated and fatigued) for the representation and the resultant TAE expected in the representation, respectively. In the proposed model (panels **a**,**d**), the tilt of the red bar on the top also illustrates adaptation in the shape and pattern representation. Blue lines illustrate the TAE observed in the psychophysical experiments. The thickness of the lines between the representations illustrates the magnitude of influence, and green and yellow denote suppressive and facilitative influence, respectively. Although all three models could reproduce the transfer condition, only the proposed model reproduced the incongruent condition (refer to the main text).

The mutual transfer between SP and SS observed in Exp. 1 supports the fact that neural circuitries for processing SP and SS operate independently, followed by the representation of “generic symmetry” that integrates SP and SS. We propose a conceptual model comprising the representations of pattern, shape, and their integration with mutual suppression between SP and SS through the formation of the integration, as shown in Fig. 6a. For instance, if the S adaptors were presented with the symmetry axis tilted to *θ*, the neural circuitry responsible for the shape representation and that responsible for the integrated representation would be activated and fatigued. A group of neurons was supposed to constitute these representations. We now consider the transfer condition when the P test stimulus is presented. Although the pattern representation was not adapted, the integrated representation was adapted, so that a certain degree of TAE is evoked (TAE_S,P_), indicating the partial transfer from SS to SP. This model with independent SS and SP processing followed by the integration reproduced the measured TAEs for all conditions in similar ways (refer to Supplementary note S5a for details). Alternative models can also reproduce the transfer from SS to SP. A model that does not distinguish between SS and SP is capable of reproducing the transfer between SS and SP (Fig. 6b). Another model in which independent SS and SP circuitries interact facilitatively with each other also reproduces the transfer without the integrated representation (Fig. 6c). However, these two models do not reproduce the prominent characteristics of the TAEs observed in the incongruent and congruent conditions, as discussed below.

In the proposed model, we assigned a stronger connection from the shape representation to the integrated representation compared to that from the pattern representation. This is based on the different magnitudes of TAE_P,P_ and TAE_S,S_. It could be argued that other types of stimuli might produce the same magnitude of effects by SP and SS and that the present difference originates from the nature of the stimuli used in the experiments. If this were the case, the weights of the connections from the shape and pattern representations would be balanced, but the essence of the model behavior would not be altered. This discussion on the unbalanced effect is also applicable to the P–S adaptor (TAE_P–S,P_ < TAE_P,P_), in which unmatched dots exist in the pattern.

A prominent characteristic of the result for incongruent adaptation (Exp. 2) is that the magnitude of TAE with the S test stimuli was similar to that observed after adaptation to SS in Exp. 1 (TAE_P–S,S_ = TAE_S,S_). Since the adaptation was incongruent where the symmetry axes were tilted in the opposite directions for SP and SS, SP and SS could cancel each other; therefore, a small degree of TAE can be expected. This prominent characteristic of TAE_P–S,S_ = TAE_S,S_ provides crucial evidence for understanding the underlying neural mechanisms. The proposed model, comprising the independent representations of pattern and shape followed by the integration, reproduces this characteristic (refer to Fig. 6d). In this model, because the representations of patterns and shapes are independent, adaptation in the shape representation is not affected by adaptation to SP. In addition, the effect of adaptation to SP in the opposite direction, which passes through the suppressive connection, operates as disinhibition and thus facilitates the effect of adaptation with SS. When the S test stimuli are presented, the adaptations in the shape and integrated representations affect the perception but not the adaptation in the pattern representation. This situation is similar to that for TAE_S,S_; therefore, the model reproduces the characteristic that TAE_P–S,S_ is similar to TAE_S,S_. Alternatively, when the P + S test stimuli are presented, the adaptations in both shape and pattern representations as well as the integrated representation affect the perception. Since the direction of the adaptation in the pattern representation is opposite to that of the shape representation, the overall magnitude of the TAE is smaller than that with the S test, leading to the reproduction of the smaller magnitude with the P + S test compared to the S test (TAE_P–S,P+S_ < TAE_P–S,S_). This model also reproduces the other TAEs with incongruent adaptation, as described in detail in Supplementary note S5b.

The mutual transfer between SP and SS observed in Exp. 1 does not exclude alternative models (Fig. 6b,c). However, these models do not reproduce the observed TAEs in incongruent conditions. First, we consider the model consisting of an integrated representation without independent pattern and shape representations (refer to Fig. 6e). After the incongruent adaptation, orientation-selective units corresponding to –θ and + θ (the tilt of SP and SS, respectively) in the integrated representation are fatigued similarly and neutralized, and thus the test stimuli produce no or small TAEs. The model response does not agree with the observed TAE with the S test stimuli, which showed a similar magnitude to the TAE with the adaptation to SS (TAE_P–S,S_ = TAE_S,S_). The reproduction of the observed TAEs must have independent pattern and shape representations. Next, we consider the model with independent pattern and shape representations with mutual facilitation between the two (refer to Fig. 6f). Since the pattern and shape representations are adapted to the opposite directions, the mutually facilitative connections work to fatigue the orientation-selective units corresponding to both directions and thus neutralize the adaptation (fatigue in both –θ and + θ); therefore, the test stimuli produce no or small TAEs. A mutually facilitative mechanism between the pattern and shape representations does not reproduce the observed TAEs.

A prominent characteristic of the result for congruent adaptation (Exp. 3) is that the TAE magnitudes were not greater than those observed after adaptation to SS or SP in Exp. 1 (TAE_P+S,S_ = TAE_S,S_; TAE_P+S,P_ = TAE_P,P_). Since the adaptation was congruent where the symmetry axes were tilted in the same direction for SP and SS, SP and SS could enhance each other; therefore, a greater degree of TAE can be expected. This characteristic is reproduced by the proposed model with mutual suppression between the pattern and shape through the formation of the integrated representation. Mutual suppression reduces the effect of adaptation in the integrated representation such that enhancement does not occur. This mutual suppression also reproduces the simultaneous reproduction of compressive and linear operations in congruent and incongruent conditions, respectively, leading to the reproduction of similar degrees of TAEs independent of adaptors (TAE_S,S_ ≈ TAE_P+S,S_ ≈ TAE_P–S,S_). With the congruent (P + S) adaptor, the suppressive connections reduce the effect of adaptation and thus enable a compressive operation. With the incongruent (P–S) adaptor, the suppressive connections act as disinhibition and facilitate the effect of adaptation, thus enabling a linear operation by overwhelming the cancelation due to the incongruence. With the SS adaptor, mutual suppression does not operate because there is no SP. The independence of TAEs from the adaptor type appears to be ecologically advantageous. The model also reproduces the other TAEs observed in the congruent condition (refer to Supplementary note S5c). The alternative models discussed in the previous paragraphs evoke a greater degree of adaptation by the congruent adaptor because they do not include suppressive mechanisms; therefore, they do not reproduce the observed TAEs.

We proposed a conceptual model based on the representations of SP and SS followed by an integrated representation, with mutual suppression between SP and SS through the formation of the integration. In reality, the representations consist of a population of neurons, and these interactions would rely on recurrent connections. It is remarkable, however, that this simple model explains the observed TAEs under all conditions (refer to Supplementary note S5). Compressive nonlinearity through mutual suppression appears to be advantageous in other integration processes. For instance, the simultaneous presentation of depth cues such as shading and texture has been known to yield an accurate perception of depth, whereas the presence of a single cue overestimates the depth^41^. Nonlinear relations with shading and highlights are also accounted for by mutual suppression^42^. It is possible that mutual suppression enables the nonlinear integration of multiple cues and achieves accurate perception.

Radial frequency (RF) patterns, stimuli defined by a sinusoidal modulation of a circle's radius as a function of the polar angle, have been reported to evoke shape aftereffects^37^. The contour shapes of our stimuli with SS somewhat resembled those of the RF patterns. It might be argued that the shape aftereffects affected the TAEs under conditions including SS in both adaptor and test, such as TAE_S,S_, TAE_P–S,S_, and TAE_P+S,S_. Lawrence et al.^37^ reported spatial variance in the shape aftereffects, suggesting that the neural mechanisms underlying the aftereffects represents a preliminary shape in the early stages of the visual cortex. In contrast, Sakata et al. reported spatial invariance in the TAE evoked by SP^35^, suggesting that the associated underlying mechanism is different from that underlying the shape aftereffects. The TAE observed with the alternative presentation of different shapes in adaptation (TAE_S-SW,S-SW_ in Supplementary note S3) also indicated insignificant shape aftereffects. It is likely that the neural mechanisms underlying the phenomena observed in the present experiments are independent of those underlying shape aftereffects. Interestingly, spatial invariance was observed in curvature-selective neurons and figure-ground-selective neurons in monkey V4^43,44^, suggesting that the neural mechanism underlying the shape aftereffect might lie earlier than V4 and that the representation of symmetry might lie in V4 or later. The results of our experiments indicated the integrated representation of symmetry of shape and pattern, suggesting the cortical representation that integrates the properties of shape and surface. The results are expected to provide crucial evidence for understanding the integration of information and representation of objects in the cortex.

## Methods

### Participants

Prior to the experiments, five individuals with normal or corrected-to-normal visual acuity gave written informed consent to participate in the experiments (age 22–25 years, one female and four males). All experiments were approved by the Research Ethics Committee of the Faculty of Engineering, Information, and Systems at the University of Tsukuba and were performed in accordance with the approved guidelines.

### Stimuli

Stimuli with SP (P stimuli) were identical to those used by Sakata et al.^35^, except for the distinct luminance within a circle that enclosed the dots (refer to Fig. 1A). Each stimulus consisted of 52 dots positioned within a circle of radius 1.5 deg. (degree of visual angle). Twenty-six dots were randomly placed on one side of the circle with respect to the vertical midline and mirrored to the other side. Each dot with a radius of 0.3 deg. (5 pixels) was randomly assigned one of eight luminance values between 81.4 and 0.5 cd/m^2^ with equal intervals. Each dot was then blurred using a Gaussian filter (SD = 3 pixels). The luminance within the enclosing circle was midgray (40.1 cd/m^2^), whereas the background luminance outside the circle was slightly darker (37.2 cd/m^2^). The dots at the circular boundary were occluded by the surrounding background (circular cutout). The occluded dots and discontinuous luminance at the circular boundary evoked a solid perception of a circular shape. Three stimuli with different dot patterns were used for the experiments.

Stimuli with SS (S stimuli) were generated similarly to those with SP, except for an egg-shaped cutout and the randomized locations of the dots without SP. The egg-shaped cutout comprised a circle with a radius of 1.5 deg. and an ellipse with minor and major radii of 1.5 and 1.94 deg., respectively. The shape was mirror symmetric with respect to the axis of SS (medial axis of the egg shape).

To examine the antagonistic interaction between SP and SS, we generated stimuli in which SP and SS were presented simultaneously with their symmetry axes tilted in the opposite directions, –10° and + 10° (anticlockwise and clockwise from the vertical), respectively, and vice versa (+ 10° and –10°, respectively; P–S stimuli; refer to Fig. 3). The outer shape was identical to that of SS, and the dot patterns were mirror symmetric with respect to the SP axis. As illustrated by the colors in Fig. 3, approximately 25% of the dots did not have a matched pair (a green region), and approximately 20% more dots constituted the pattern symmetry (a light-blue region) than in the P stimuli. To examine the synergic interaction, we generated stimuli in which the symmetry axes for SP and SS were aligned and tilted in the same direction where the tilt was either –10° or + 10° (P + S stimuli). The stimuli were identical to those with P–S, except for the tilt direction of the symmetry axes. When both adaptors and test stimuli included SP (such as in TAE_P,P_, TAE_P–S,P_, and TAE_P–S,P+S_), the luminance contrasts of dots in the test stimulus were inverted to cancel out the adaptation to contrast. In the case of TAE_S,S_, the adaptors and test stimuli had different dot patterns.

### Procedure and apparatus

The procedure was adopted from Sakata, et al.^35^ A pair of stimuli were shown, one above and one below the fixation (an example is illustrated in Fig. 1B). During each session, the participants were instructed to gaze at the fixation aid at the center of the screen. Each session began with an initial adaptation period of 60 s, followed by a repeated test of 0.5 s duration interspersed with top-up adaptation periods of 2.0 s durations to reinforce the initial adaptation. During the initial and top-up adaptation periods, a pair of stimuli were presented simultaneously. The invisible symmetry axes of the top and bottom adaptors were oriented at − 10° and + 10°, respectively, from the vertical direction, or + 10° and − 10°, respectively, and their tilt remained constant during a session. It has been reported that adaptation to low-level features, such as retinal position and luminance contrast, slightly increased the TAE magnitude^31,33,35^. To prevent adaptation to the low-level image properties, the adaptors were rotated along an imaginary circle with the tilt of their symmetry axes remaining constant. Specifically, the pair of adaptors were rotated in the opposite directions along imaginary circles of radius 1 deg. at 0.5 rev/s with centers positioned 3 deg. above or below the fixation aid. With the circular stimuli (P), the stimulus center moved along the imaginary circle, while with the egg-shaped stimuli (S, P–S, and P + S), the center of the circular portion moved along the circle. These rotating adaptors were not expected to evoke the perception of an imaginary shape, afterimage, or sustained responses of particular neurons in the early visual areas. Since it has been reported that symmetry detection is independent of motion and dependent on the lifetime of the dots (equivalent to the number of dots)^16^, it is unlikely that the motion in our stimuli altered the perception of symmetry.

During the test period (0.5 s), a pair of stimuli distinct from those used in the adaptation period was presented. The color of the fixation aid was changed to green during the test period. The centers of the test stimuli were positioned 3 deg. above and below the fixation aid. A staircase method with a two-alternative forced-choice was employed to measure the TAE in accordance with Gheorghiu et al.^45^ The stimuli were presented with their axes upright in the first trial. During each test trial, the participants were asked to indicate which stimulus (top or bottom) appeared tilted clockwise. Upon response, top-up adaptation was initiated. Based on the participant's answer, the next stimuli were rotated so that the TAE was canceled, as stipulated by the staircase method. The trial was repeated 25 times, and the stimuli were rotated at 1° for the first five trials and 0.5° subsequently. The magnitude of the TAE was defined as the mean angular difference between the pair of test stimuli over the last 20 trials, which typically included 4–13 reversals (mean = 8.4). We used a method that was terminated after a fixed number (25) of trials rather than a fixed number of reversals. This method is advantageous because it maintains as constant the total amount of adaptation for each condition. At the beginning of the experiment, participants carried out five to ten practice sessions to familiarize themselves with the procedure and fixation. Their eye movements were monitored (X2-60, Tobii, Sweden), and participants who moved their eyes were instructed to keep them fixed.

The participants sat 1.0 m from a display in a dark room. Stimuli were presented on the monitor with γ = 1.0 at a refresh rate of 60 Hz (Color Edge CG243 W-B, EIZO, Japan). The distance between the center and the side edge of the monitor was 16.5 deg., which ensured that the edges had a negligible influence on the experimental results. The experiments were controlled by custom software written in the C programming language and OpenGL running on a Mac Pro (Apple, CA, U.S.A.). R software (version 3.6.2; https://www.R-project.org) was used for statistical analysis. Prior to the parametric tests, we tested for normality (one-sample Kolmogorov–Smirnov test) and homogeneity in variance (*F*-test and Bartlett test). If either test was significant (α = 0.01), we performed a randomization test (RVAideMemoire package for R) in addition to the parametric test and confirmed that the results were consistent between the tests. In pairwise *t*-tests, data were paired based on the participant, stimulus, and test order. The results of the pairwise *t*-tests were consistent with one-way within-subject ANOVA. All statistical tests were two-sided.

## Supplementary Information

Supplementary Information
