# Supplementary material for: Interaction of surface pattern and contour shape in the tilt after effects evoked by symmetry
Source: Sci Rep. 2021 Apr 13;11:8024. doi: 10.1038/s41598-021-87429-y (PMC8044203; doi:10.1038/s41598-021-87429-y)
Supplement: Supplementary file 1 — Supplementary Information [file 41598_2021_87429_MOESM1_ESM.pdf]

SUPPLEMENTARY INFORMATION

Interaction of Surface Pattern and Shape in the Tilt After Effects Evoked by Symmetry

Ko Sakai*), Yui Sakata, and Ken Kurematsu

Supplement 1. Blank condition

To examine the exact amount of TAE evoked by adaptation, we measured the perceptual tilt of the symmetry axis in the test stimuli without adaptation (blank condition: TAB_B). A blank screen with midgray was presented during the initial and top-up adaptation periods. The stimuli and experimental procedure were identical to those used in the main text. The measured tilts of the P, S, and P+S test stimuli were 0.398°, 0.435°, and 0.018°, respectively, as shown in Fig. 2 (P and S test) and Fig. 5 (P+S test). The measured tilt was not significantly different from zero (one-sample *t*-test; P test: $t(59) = 1.75$, $p = 0.086$; S test: $t(59) = 1.35$, $p = 0.182$; P+S test: $t(59) = 0.098$, $p = 0.922$). These results indicate that the test stimuli evoked the perception of tilt, similar to the geometrically defined tilt.

Supplement 2. Statistical analysis

p, adjusted: p-value obtained using a pairwise t-test with Bonferroni correction.
p, random: p-value obtained by a pair-wise randomization test with Bonferroni correction. A randomization test was performed when normality and/or equal variance were violated.

Table S2-1 Multiple comparisons among the four conditions in Exp.1

conditions			t(59)	p, adjusted	p,random	significance
$TAE_{p,p}$	v.s.	$TAE_{p,s}$	1.31	1.00	1.00	
$TAE_{p,p}$	v.s.	$TAE_{s,p}$	0.396	1.00	1.00	
$TAE_{p,p}$	v.s.	$TAE_{s,s}$	7.74	<0.001	0.001	***
$TAE_{p,s}$	v.s.	$TAE_{s,p}$	1.58	0.714	1.00	
$TAE_{p,s}$	v.s.	$TAE_{s,s}$	6.66	<0.001	0.001	***
$TAE_{s,p}$	v.s.	$TAE_{s,s}$	8.99	<0.001	0.001	***

Table S2-2 Comparisons with blank conditions in Exp.2

	t(59)	p	p, random
$TAE_{p\text{-}s,p}$	0.441	0.661	0.66
$TAE_{p\text{-}s,p+s}$	4.61	<0.001	-
$TAE_{p\text{-}s,s}$	7.51	<0.001	<0.001

Table S2-3 Multiple comparisons among the three conditions in Exp.2

conditions			t(59)	p, adjusted	p, random	significance
$TAE_{p\text{-}s,p}$	v.s.	$TAE_{p\text{-}s,p+s}$	4.58	<0.001	<0.001	***
$TAE_{p\text{-}s,p}$	v.s.	$TAE_{p\text{-}s,s}$	9.66	<0.001	<0.001	***
$TAE_{p\text{-}s,p+s}$	v.s.	$TAE_{p\text{-}s,s}$	7.26	<0.001	<0.001	***

Table S2-4 Comparisons with blank conditions in Exp.3

	t(59)	p	p, random
$TAE_{p+s,p}$	5.25	<0.001	-
$TAE_{p+s,p+s}$	10.3	<0.001	-
$TAE_{p+s,s}$	10.3	<0.001	<0.001

Table S2-5 Multiple comparisons among the three conditions in Exp.3

conditions			t(59)	p, adjusted	p, random	significance
$TAE_{p+s,p}$	v.s.	$TAE_{p+s,p+s}$	3.12	0.008	0.003	**
$TAE_{p+s,p}$	v.s.	$TAE_{p+s,s}$	5.26	<0.001	<0.001	***
$TAE_{p+s,p+s}$	v.s.	$TAE_{p+s,s}$	3.32	0.005	0.007	**

Supplement 3. Control experiment for TAE evoked by shape symmetry

To examine whether the stimuli with SS in fact evoked TAE, we measured TAE for two control conditions. First, we tested the effect of randomly placed dots within the egg-shaped contour by removing the dots from the SS stimuli used in Exp. 1 ($TAE_{S\text{-}ND,\ S\text{-}ND}$). Second, we tested the effect of shape afterimage by alternatively presenting two stimuli (the original SS stimulus and its upside-down image; ($TAE_{S\text{-}SW,\ S\text{-}SW}$)). The procedure was identical to those in Exp.1. Four participants (one female and three male) carried out the experiment; all of them were different from those performed Exp. 1 to 3, but the procedures and attributes were identical to those described in the Participants section in the main text.

The mean measured TAE magnitudes across the participants are shown in Fig. S3-1, together with the result of SS condition in Exp.1 ($TAE_{S,\ S}$). To examine whether the measured magnitudes between $TAE_{S,\ S}$ and the other conditions ($TAE_{S\text{-}ND,\ S\text{-}ND}$, $TAE_{S\text{-}SW,\ S\text{-}SW}$) were different, we performed permutation tests with Bonferroni correction since equal variance was violated. The results did not show significant differences between any two conditions ($TAE_{S,\ S} – TAE_{S\text{-}ND,\ S\text{-}ND}$: $p = 0.24$; $TAE_{S,\ S} – TAE_{S\text{-}SW,\ S\text{-}SW}$: $p = 1.00$; $TAE_{S\text{-}ND,\ S\text{-}ND} – TAE_{S\text{-}SW,\ S\text{-}SW}$: $p = 0.11$). These results indicate that the dots within the egg-shaped contour and the shape afterimage did not significantly influence the measured TAE magnitude.

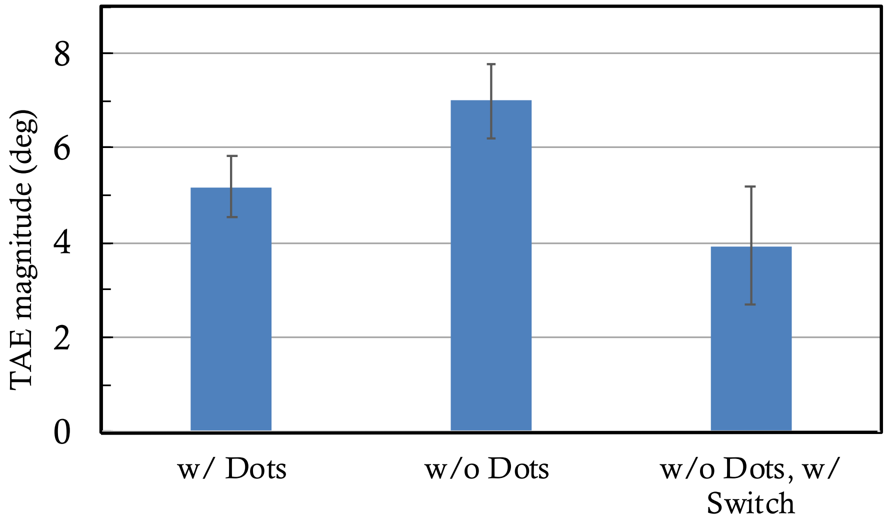

Fig. S3-1. The mean TAE magnitudes across participants for the three conditions. From the left, $TAE_{S, S}$, $TAE_{S\text{-}ND, S\text{-}ND}$, and $TAE_{S\text{-}SW, S\text{-}SW}$. Error bars indicate SEM. We observed no significant difference between $TAE_{S, S}$ and the other conditions ($TAE_{S\text{-}ND, S\text{-}ND}$ and $TAE_{S\text{-}SW, S\text{-}SW}$).

Supplement 4. Individual data for Exp.2 and 3

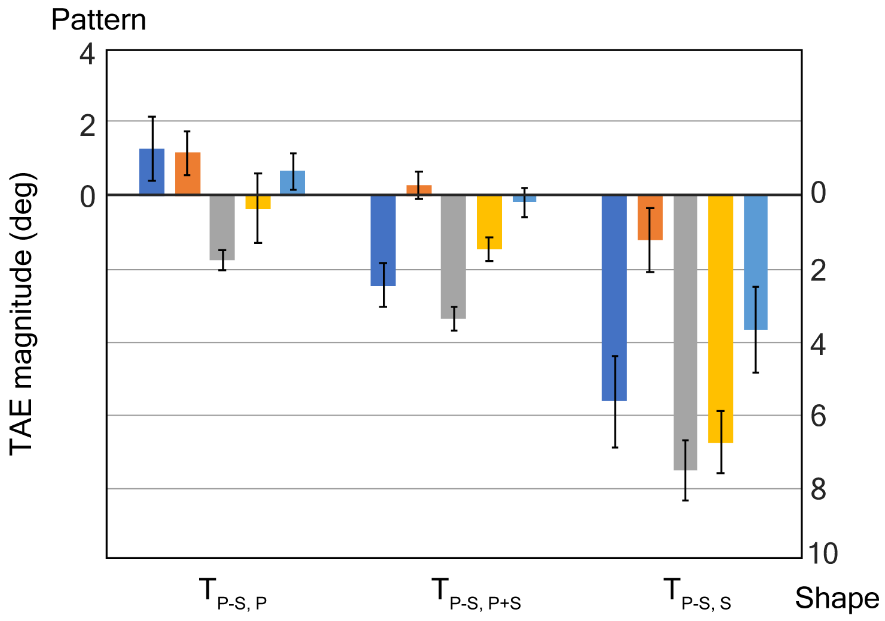

Fig. S4-1. The measured magnitude of each participant with incongruent condition (Exp.2). Colors indicate individuals. The TAE for SP is plotted toward the positive direction in the ordinate (the scale on the left) whereas the TAE for SS is plotted towards the negative direction (the scale on the right). Error bars show the standard error (SEM).

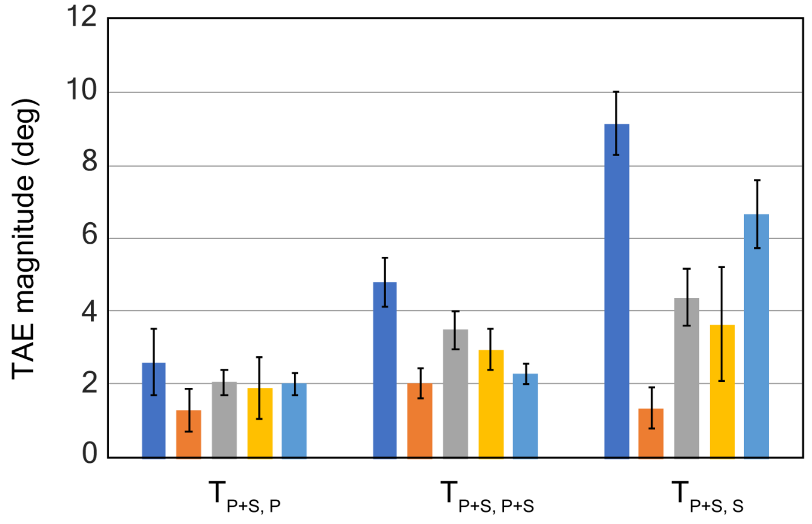

Fig. S4-1. The measured magnitude of each participant with the congruent condition (Exp.3). Colors indicate individuals. Error bars show the standard error (SEM).

Supplement 5. The responses of the model

We propose a conceptual model comprising the representations of pattern, shape, and their integration with mutual suppression between SP and SS through the formation of the integration, as described in the Discussion section. This supplementary note describes the responses of the model that qualitatively reproduces the observed TAEs under all conditions.

(a) Responses to the transfer conditions (Exp.1)

The proposed model reproduces the observed TAEs in the control and transfer conditions (Exp. 1; Fig. S5-1).

Reproduction of $TAE_{S,\,S}$ --- control condition

When an adaptor with SS (S adaptor, the red bar at the top of panel (a)) is presented with the symmetry axis tilted to θ, neural circuits responsible for the shape representation and that responsible for the integrated representation are activated (as illustrated by the red bars next to the representations) and fatigued. When a test stimulus with SS (S test, the green bar at the top of the panel (a)) is then presented, both the shape and integrated representations exhibit TAE in the opposite direction to the symmetry axis of the adaptor (the light-blue bars, tilted to $-\theta$). The perception of the tilt (the blue bar; $TAE_{S,\,S}$) depends on the tilt of the integrated representation. For the sake of simplicity, we describe here that the TAE ($-\theta$) is the opposite to the tilt of adaptor (θ). $TAE_{P,\,P}$ is explained similarly (refer to the panel (c)) but with a smaller magnitude of TAE because of a weaker connection from the pattern representation to the integrated representation.

Reproduction of $TAE_{S,\,P}$ --- transfer condition

We consider transfer condition with the S adaptor and P test (the red and green bars at the top of panel (b), respectively). With the S adaptor, the shape and integrated representations are activated and adapted (the red bars next to the representations) but not the pattern representation. When the P test is then presented, no TAE is evoked in the pattern representation. Although the pattern representation is not adapted, the integrated representation is adapted, so that a smaller degree of TAE is observed compared to that measured with the S test ($TAE_{S,\,P} < TAE_{S,\,S}$), indicating the partial transfer from SS to SP.

(b) Responses to the incongruent conditions (Exp.2)

The proposed model reproduces the observed TAEs evoked by the incongruent adaptors (Exp.2; Fig. S5-2).

Reproduction of $TAE_{P\text{-}S,\,P}$

With the P test stimuli, the measured magnitude of $TAE_{P\text{-}S,\,P}$ was significantly smaller than that of $TAE_{P,\,P}$ in Exp. 1. The model reproduces this observation as shown in panel (a). Under the condition of $TAE_{P\text{-}S,\,P}$, the directions of TAE in the pattern and integrated representations are opposite because the directions of adaptation are opposite in SP and SS, and the SS is dominant through the integration of SP and SS. When the P test stimulus is presented, the pattern representation yields TAE with a magnitude similar to $TAE_{P,\,P}$. However, it is then canceled and the TAE in the integrated representation is rather biased toward the SS, resulting in $TAE_{P\text{-}S,\,P} < TAE_{P,\,P}$.

Reproduction of $TAE_{P\text{-}S,\,S}$

With the S test stimuli, the measured magnitude of $TAE_{P\text{-}S,\,S}$ was smaller than that of $TAE_{S,\,S}$ in Exp. 1, but the difference was not statistically significant. The model reproduces this observation as shown in panel (b). Since $TAE_{P\text{-}S,\,S}$ and $TAE_{S,\,S}$ share the S test stimuli and the adaptation in the shape and integrated representations (compare the panel (b) and S5-1(a)), similar degrees of TAEs are expected ($TAE_{P\text{-}S,\,S}$ = $TAE_{S,\,S}$). Note that the adaptation by the P–S adaptor in the integrated representation is considered to be similar to or slightly weaker than the adaptation by the S adaptor despite the incongruent adaptation. This is because the mutual suppression between the SP and SS through the formation of the integrated representation acts as disinhibition.

Reproduction of $TAE_{P\text{-}S,\,P+S}$

With the P+S test stimuli, the measured magnitude of $TAE_{P\text{-}S,\,P+S}$ was significantly smaller than that with the S test stimuli ($TAE_{P\text{-}S,\,S}$; refer to the panel (b)). The model reproduces this observation as shown in panel (c). When the P+S test is presented, the pattern and shape representations produce TAEs similar to $TAE_{P,\,P}$ and $TAE_{S,\,S}$, respectively, in opposite directions. Therefore, they cancel each other in the integrated

representation. The cancelation leads to a smaller magnitude in $TAE_{P\text{-}S,\,P+S}$ than in $TAE_{P\text{-}S,\,S}$ ($TAE_{P\text{-}S,\,P+S} < TAE_{P\text{-}S,\,S}$).

(b) Responses to the congruent conditions (Exp.3)

The proposed model reproduces the observed TAEs evoked by the congruent adaptors (Exp.3; Fig. S5-3).

Reproduction of $TAE_{P+S,\,P}$ and $TAE_{P+S,\,S}$

With the S test stimuli, the measured magnitude of $TAE_{P+S,\,S}$ was not significantly greater than $TAE_{S,\,S}$ (refer to Fig. S5-1(a)). The model reproduces this observation as shown in panel (b). Since the pattern and shape representations share the same direction of adaptation (θ; as illustrated by the red bars next to the representations), it might be argued that the degree of adaptation in the integrated representation could be increased. However, the mutual suppression between the SP and SS in our model prevents the increase of adaptation in the integrated representation as illustrated by the red bar next to the integrated representation. Note that the degrees of adaptation in the shape and integrated representations are identical to those evoked by the S adaptor. Therefore, when the S test is presented, a similar magnitude of $TAE_{P+S,\,S}$ is evoked compared to $TAE_{S,\,S}$. Note also that, during the presentation of test stimuli, the suppression operates effectively only when both SP and SS are present as with the P+S test but not with the S test. The mutual suppression through the formation of the integrated representation contributes to the reproduction of $T_{P+S,\,S} = T_{S,\,S}$. Similarly, the model reproduces $TAE_{P+S,\,P} = TAE_{P,\,P}$, as shown in the panel (a).

Reproduction of $TAE_{P+S,\,P+S}$

With the P+S test, the measured magnitude of $TAE_{P+S,\,P+S}$ was significantly smaller than that with the S test, $TAE_{P+S,\,S}$. The model also reproduces this observation as shown in panel (c). When the P+S test is presented after the adaptation, unlike the S test, the signals from both pattern and shape representations come into the integrated representation, so that the suppression operates effectively; thus, a smaller degree of TAE is evoked compared to that with the S test ($TAE_{P+S,\,P+S} < TAE_{P+S,\,S}$).

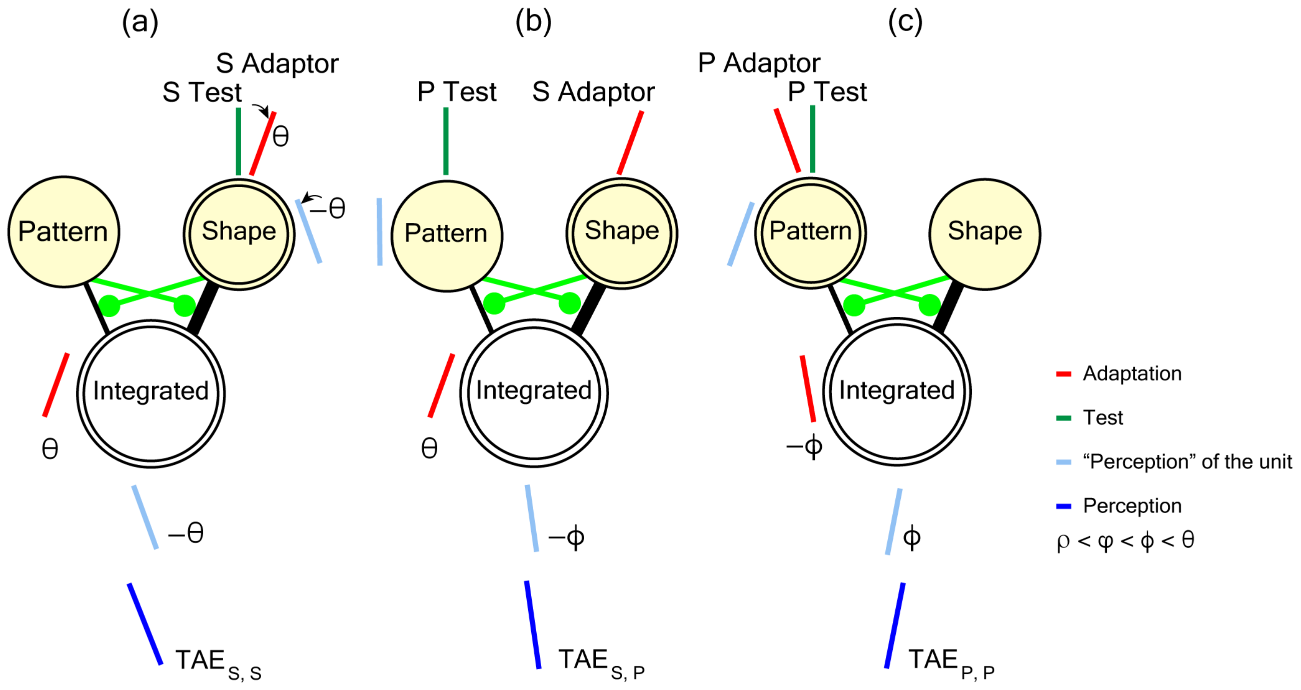

Fig. S5-1.

An illustration of the responses of the proposed model with the control and transfer conditions (Exp.1). The model and conventions are the same as those in Fig. 6 in the main text. **(a)** Control condition with the S adaptor (tilted to θ) and S test. The red and green bars at the top illustrate the adaptor and test, respectively. The red bar also illustrates adaptation in the shape representation, *i.e.*, neurons responsive to this tilt are activated and fatigued. A light-blue bars on the right of the shape representation illustrates the expected TAE in the representation ($-\theta$) resulting from the adaptation. The light-blue bar at the bottom of the integrated representation also illustrates the expected TAE in the representation. The blue bar at the bottom shows the TAE measured by psychophysical experiments. **(b)** Transfer condition with the S adaptor and P test. This panel is identical to that shown in Fig.6a. **(c)** Control condition with the P adaptor and P test. As illustrated by the thickness of the lines between the representations, the shape representation has a greater influence than the pattern representation through the formation of the integrated representation; thus, $TAE_{P,P}$ is smaller than $TAE_{S,S}$. The model reproduces the observed relations among TAEs in magnitude, $TAE_{S,S} > \{TAE_{S,P}, TAE_{P,P}\}$, and in direction, $\{TAE_{S,S}, TAE_{S,P}\}$ vs. $TAE_{P,P}$.

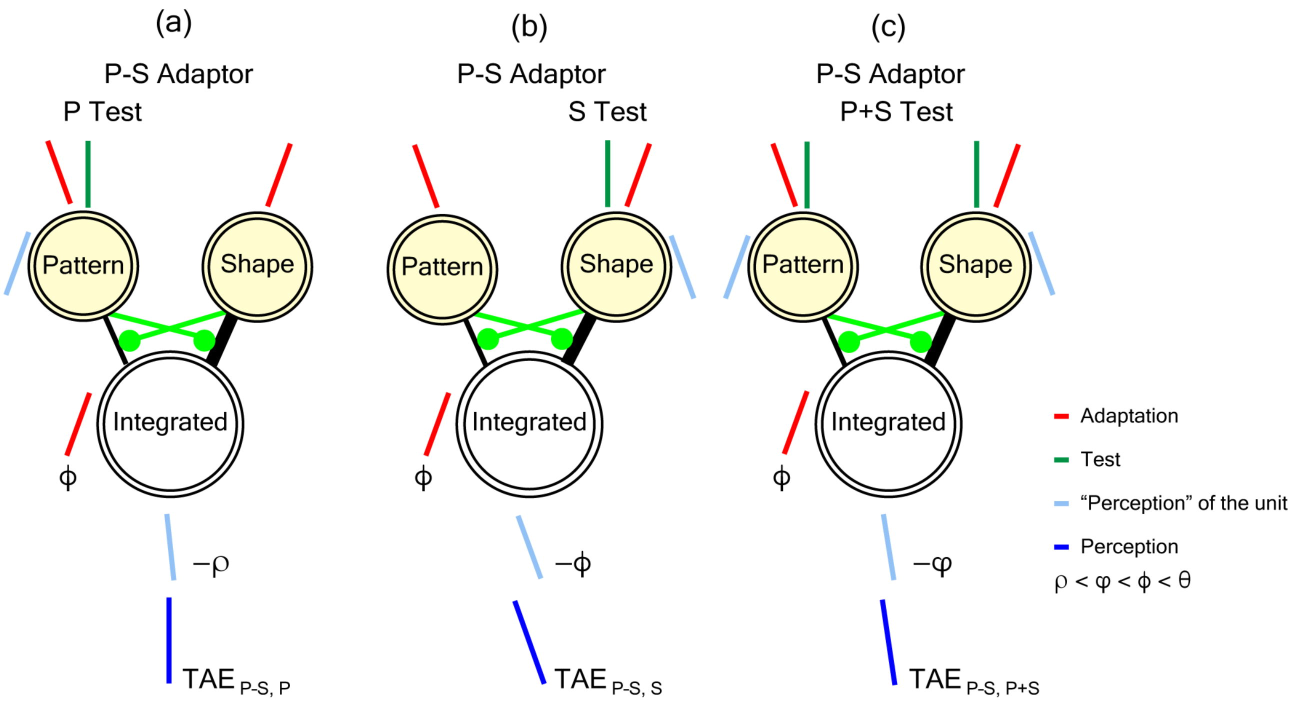

Fig. S5-2

The responses of the proposed model with the incongruent adaptors (P–S adaptor in Exp 2). The model and conventions are the same as those in Fig. S5-1. Panels **a**, **b**, and **c** illustrate the model responses for $TAE_{P–S,\,P}$, $TAE_{P–S,\,S}$, and $TAE_{P–S,\,P+S}$, respectively. The red and green bars illustrate the adaptors and test stimuli. The blue bars at the bottom illustrate the TAE observed in the psychophysical experiment. The model reproduces the relation of TAE magnitude, $TAE_{P–S,\,S} > TAE_{P–S,\,P+S} > TAE_{P–S,\,P}$.

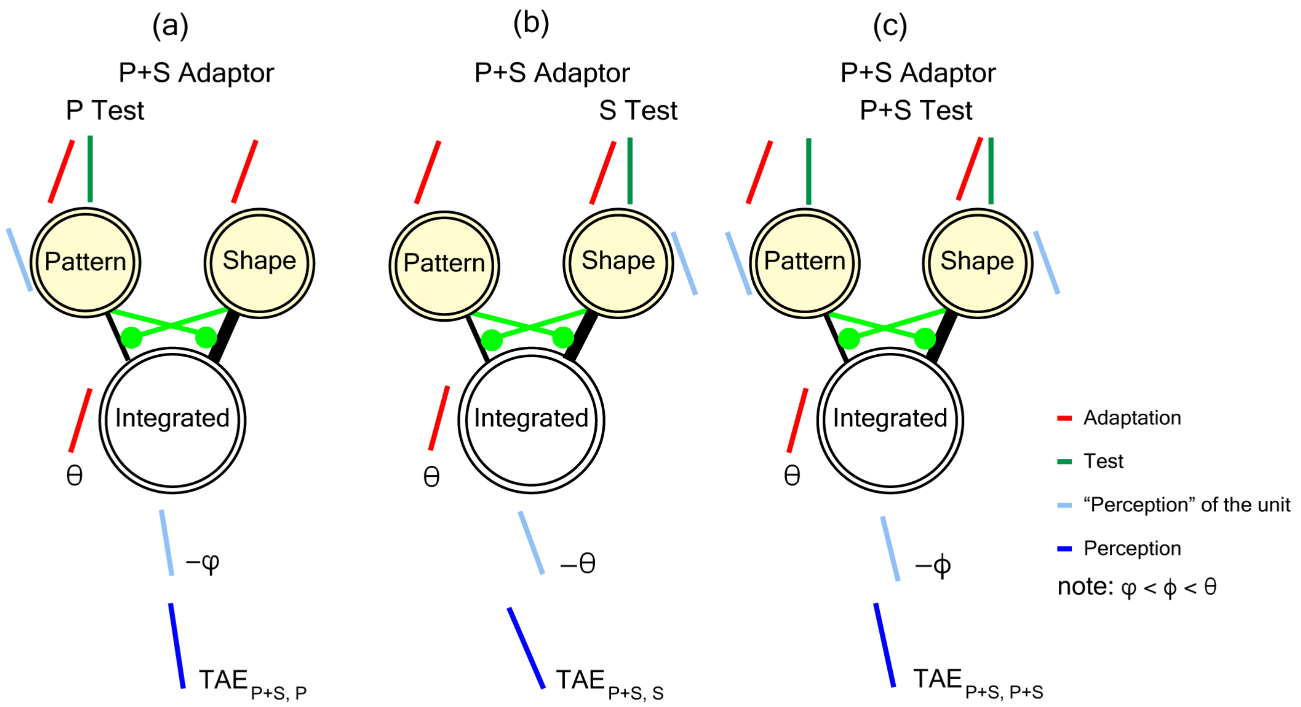

Fig. S5-3

The responses of the proposed model with the congruent adaptors (P+S adaptor in Exp.3). The same conventions are used as in Fig. S5-1. Panels **a**, **b**, and **c** show the $TAE_{P+S,\,P}$, $TAE_{P+S,\,S}$, and $TAE_{P+S,\,P+S}$, respectively. The model reproduces the relation in TAE magnitude, $TAE_{P+S,\,S} > TAE_{P+S,\,P+S} > TAE_{P+S,\,P}$.
